# Medical Child Abuse: A Retrospective Analysis from a Tertiary Pediatric Hospital’s Childhood and Adolescent Abuse Group

**DOI:** 10.3390/children12111575

**Published:** 2025-11-20

**Authors:** Martina Focardi, Marta Guerini, Beatrice Defraia, Laura Nanni, Rossella Grifoni, Giovanni Castellini, Barbara Gualco, Ilenia Bianchi, Vilma Pinchi, Stefania Losi

**Affiliations:** 1 Section of Forensic Medical Sciences, Department of Health Sciences, University of Florence, 50134 Florence, Italy; martina.focardi@unifi.it (M.F.); marta.guerini@unifi.it (M.G.); rossella.grifoni@unifi.it (R.G.); barbara.gualco@unifi.it (B.G.); ilenia.bianchi@unifi.it (I.B.); vilma.pinchi@unifi.it (V.P.); 2 GAIA Service (Childhood and Adolescent Abuse Group), AOU Meyer IRCCS, 50139 Florence, Italy; laura.nanni@meyer.it (L.N.); stefania.losi@meyer.it (S.L.); 3 Section of Psychiatry, Department of Health Sciences, University of Florence, 50100 Florence, Italy; giovanni.castellini@unifi.it

**Keywords:** medical child abuse, Münchausen syndrome by proxy, fabricated or induced illness, child maltreatment, forensic pediatrics, perplexing presentations, factitious disorder imposed on another

## Abstract

Background: Medical child abuse (MCA), previously known as Münchausen syndrome by proxy, involves the fabrication or induction of illness by caregivers—most commonly the mother—leading to unnecessary medical interventions and potential harm to the child. Methods: This retrospective study analyzed cases of suspected or confirmed MCA managed by the GAIA multidisciplinary team at Meyer Children’s Hospital, Florence, between 2010 and 2022. Cases were identified using Rosenberg diagnostic criteria and red flags outlined by the American Academy of Pediatrics (AAP) and the Royal College of Paediatrics and Child Health (RCPCH). Data were extracted from medical records and analyzed descriptively. Results: Among 816 cases of child maltreatment, 8 (0.99%) were identified as MCA. The median age of affected children was 5 years (range: 4–12 years), with a female predominance (6/8, 75%). All perpetrators were biological mothers (8/8, 100%). Children had a median of 23 emergency department visits (range: 4–44), with the most frequent presentations being fever (6/8, 75%), minor trauma (7/8, 87.5%), respiratory complaints (5/8, 62.5%), and gastrointestinal symptoms (4/8, 50%). According to Rosenberg criteria, 5 cases (62.5%) were classified as “possible diagnosis,” 1 (12.5%) as “definitive diagnosis,” 1 (12.5%) as “diagnosis by exclusion,” and 1 (12.5%) as “inconclusive.” Conclusions: Despite its low prevalence, MCA poses serious clinical and ethical challenges. Early detection requires thorough documentation, interdisciplinary collaboration, and improved access to shared medical records. The GAIA model offers a replicable framework for effective multidisciplinary management.

## 1. Introduction

Münchausen syndrome by proxy (MSbP) has been reclassified in the DSM-5 as factitious disorder imposed on another (FDIA), reflecting a more precise understanding of this uncommon but serious form of child abuse [1]. The perpetrator—not the child—is assigned the diagnosis of FDIA.

The term ‘syndrome’ derives from Baron Münchhausen, an 18th-century historical figure renowned for his remarkable and fictitious narratives, and was originally employed to describe patients who feigned illnesses in themselves [2,3]. The ‘proxy’ variant was first described in 1977 by British pediatrician Sir Roy Meadow, who identified a pattern in which caregivers—typically mothers—fabricated or induced illness in their children to gain attention or sympathy from medical professionals [4].

Early clinical literature positioned MSbP primarily as a psychiatric disorder in the caregiver, often linked to personality disorders or histories of abuse. This caregiver-focused framing led to a strong interest in psychological profiles, though it also sparked controversy due to difficulties in establishing motive and the high stakes of misdiagnosis.

Throughout the 1980s and 1990s, the diagnosis of MSbP grew in both prominence and controversy. As Rosenberg (1987) outlined in a widely cited review, the condition was characterized by a triad: falsification of medical history, induction of symptoms, and persistent medical-seeking behavior [5].

However, critics argued that too much emphasis was placed on parental psychopathology, which was difficult to prove and often relied on subjective clinical judgment. Moreover, early diagnostic frameworks lacked consistent criteria, leading to a broad—and at times overly flexible—application of the label [6].

In response to these challenges, a shift in nomenclature and clinical framing emerged in the early 2000s. In 2007, the American Academy of Pediatrics (AAP) issued guidance advocating the use of the term “medical child abuse” (MCA) [7]. This reframing aimed to emphasize the objective harm to the child—whether by fabrication, exaggeration, or induction of illness—rather than any presumed psychological disorder in the caregiver.

Following suit, the Royal College of Paediatrics and Child Health (RCPCH) in the UK published its own recommendations in 2009, introducing two new terms: fabricated or induced illness (FII), denoting situations in which the caregiver actively misleads clinicians or causes illness in a child; and perplexing presentations (PP), referring to cases where the child’s clinical picture is inconsistent or difficult to explain but may not meet the full criteria for FII [8].

These revisions aimed to improve early identification, reduce harm, and allow for graded interventions based on risk and evidence, without requiring a psychiatric diagnosis of the caregiver.

Recent literature has continued to explore the spectrum of behaviors that fall under medical child abuse, emphasizing interdisciplinary collaboration among pediatrics, psychiatry, child protection, and legal professionals. Research by Stirling et al. (2013) has shown that effective management often requires a multifactorial approach, including covert video surveillance in hospital settings, psychological assessments, and careful documentation [9].

At the same time, ethical concerns persist, particularly around surveillance methods, the potential for misidentifying medically complex conditions as abuse, and the long-term psychological effects on families wrongly accused. This has led some scholars to call for standardized risk assessment tools, improved clinician training, and better legal safeguards to balance child protection with parental rights.

Child abuse remains a major global concern. The World Health Organization (WHO) estimates that 1 in 4 adults experienced physical abuse in childhood, and 1 in 5 women experienced childhood sexual abuse [10]. MCA is a rare but highly dangerous form of abuse, with an estimated incidence of 0.5 to 2 per 100,000 children annually, and mortality rates ranging from 6–10% [11,12,13,14]. Many survivors suffer long-term physical and psychological harm. The average age of affected children is under 6 years, and mothers are the perpetrators in more than 85–95% of confirmed cases. The duration of abuse before detection often exceeds 1 year, typically involving multiple healthcare contacts [11,12,13,14].

### 1.1. Terminology and Diagnostic Framework

Throughout this paper, we use “medical child abuse” (MCA) as the primary term, consistent with AAP guidelines, while acknowledging related terminology:

- MSbP (Münchausen syndrome by proxy): Historical term emphasizing caregiver psychopathology
- FDIA (factitious disorder imposed on another): DSM-5 psychiatric diagnosis assigned to the perpetrator
- FII (fabricated or induced illness): UK term focusing on caregiver behavior
- PP (perplexing presentations): Cases with inconsistent clinical pictures not meeting full FII criteria [11,12,13,14].

### 1.2. Perpetrator Characteristics

The majority of perpetrators are primary caregivers, usually the biological mother. Several psychological and psychosocial characteristics have been noted in the majority of perpetrators (Table 1):

These individuals often appear overinvolved, cooperative, and medically knowledgeable, which may delay suspicion from healthcare providers [16,17].

Dysfunctional family dynamics and stressors can create a context in which MCA emerges as marital conflict or domestic violence, absent or passive second caregiver (e.g., disengaged father), financial or emotional stress, frequent relocation or hospital-hopping to avoid detection, or previous involvement with child protection services.

### 1.3. Victim Characteristics

Children targeted in MCA are often young, vulnerable, and less able to communicate their symptoms clearly. In the table below, the main risk factors of the victims are summarized (Table 2).

### 1.4. Diagnostic Criteria

Rosenberg [19] provides a hierarchy of diagnostic conviction (definitive, possible, inconclusive, definitely excluded) and lists core features, which are condensed here:

a Definitive diagnosis: -Fabrication or induction of physical or psychological signs or disease in a child by a caregiver; -Presentation of the child by the caregiver as ill, impaired or injured; -Deceptive behavior by the caregiver is evident (i.e., intent to deceive); -The child has undergone multiple medical evaluations, treatments, hospitalizations, often unnecessary or harmful; -The behavior continues despite negative findings or absence of disease; -There is no obvious external reward (e.g., financial gain) that better explains the behavior; -Alternative explanations (e.g., genuine disease, other forms of abuse) have been considered and excluded as a better explanation.
b Possible diagnosis: -Core behavior present but some major elements missing (e.g., less evidence of deception, fewer hospitalizations)—diagnostic certainty lowered.
c Inconclusive/definitely excluded: Inconclusive: Insufficient data to confirm or exclude. -Definitely excluded: Clear evidence points to another cause (e.g., genuine illness, other abuse) or no caregiver deception.

The American Academy of Pediatrics (AAP) [7] and Royal College of Paediatrics and Child Health (RCPCH, UK 2009) [8] do not provide a rigid checklist of criteria in the same way Rosenberg does, but they do identify key features/alerting signs [20]. 

In the Table 3 below we summarize the key diagnostic red flags reported by AAP and RCPCH:

Frequent interactions with various healthcare facilities, consultations with multiple specialists, and a disjointed or poorly substantiated clinical history are commonly noted.

The techniques for falsifying or inducing symptoms are numerous and encompass simulation, omission, exaggeration, pharmacological or physical symptom induction, and manipulation of biological samples and clinical records. The primary symptoms that are induced include fever, allergic conditions, epilepsy, factitious bleeding, renal or gastrointestinal disorders, factitious dermatitis, and respiratory illnesses [21]. The increasing focus on this condition arises not only from the severity of its physical and psychological repercussions on victims, but also from the difficulties related to its detection and clinical management [22,23].

The AAP emphasizes a multidisciplinary evaluation involving pediatricians, mental health professionals, social services, and legal authorities, while RCPCH emphasizes the use of covert video surveillance (CVS) in hospital settings under strict ethical and legal oversight (in rare but severe cases) and the importance of record-keeping, team consensus, and safeguarding procedures.

### 1.5. Study Objective

This retrospective study aims to do the following:

1. Determine the prevalence of MCA among child maltreatment cases at a tertiary pediatric hospital;
2. Characterize the demographic and clinical profiles of affected children;
3. Describe perpetrator characteristics and patterns of healthcare utilization;
4. Evaluate the effectiveness of the GAIA multidisciplinary approach in MCA diagnosis and management.

## 2. Materials and Methods

### 2.1. Study Design and Setting

This retrospective case series was conducted at Meyer’s Children Hospital, a tertiary pediatric hospital in Florence, Italy. The study was approved by the hospital’s ethics committee (code: n.49/2023_oss, date: 21 February 2023) and conducted in accordance with the Declaration of Helsinki.

### 2.2. Study Population

We reviewed all cases of suspected or confirmed MCA managed by the GAIA service (Childhood and Adolescent Abuse Group; https://www.meyer.it/cura-e-assistenza/attivita-sanitarie/590-sportello-gaia (accessed on 11 January 2025)) from 1 January 2010 to 31 December 2022. The GAIA service consists of a multidisciplinary team including child abuse pediatricians, forensic pathologists, child neuropsychiatrists, psychologists, nurses, and social workers dedicated to child protection.

**Inclusion criteria** were all cases of suspected or confirmed MCA identified by the GAIA team during the study period with complete medical records available for review.

**Exclusion criteria** were insufficient documentation to apply diagnostic criteria.

### 2.3. Case Definition and Diagnostic Criteria

The diagnosis of MCA was made according to Rosenberg diagnostic criteria [19] and AAP and RCPCH red flags [7,8], with particular emphasis placed on the following:

- Illnesses that were unexplained or prolonged;
- Symptoms and signs that appeared inappropriate or incongruous;
- Symptoms that only manifested in the presence of a parent (typically the mother);
- Treatments that proved ineffective or were poorly tolerated;
- Family histories of sudden unexplained infant deaths;
- Families with multiple members purported to have various serious medical conditions.

Maternal behavior was scrutinized for suspicious patterns, including lack of concern regarding the child’s illness, constant presence at the child’s bedside without breaks, or development of close relationships with medical staff.

A differential diagnosis was meticulously conducted to rule out genuine organic diseases and malingering behavior.

### 2.4. Gaia Multidisciplinary Protocol

The GAIA team follows a standardized nine-step protocol:

1. Medical history review: Child abuse pediatricians conduct a thorough and comprehensive medical history, corroborated by data retrieved from previous hospital encounters. When feasible, caregivers are interviewed separately, and the child is interviewed directly.
2. Psychological assessment: A psychologist and a child neuropsychiatrist carry out an initial psychological and psychiatric assessment of both the child and the caregiver.
3. Physical examination: Child abuse pediatricians perform a detailed physical examination, documenting any somatic injuries, and correlating these findings with both the clinical and reported histories.
4. Diagnostic investigations: Child abuse pediatricians prescribe appropriate diagnostic investigations, including radiological imaging, laboratory blood tests, and toxicological screening.
5. Specialist consultations: Child abuse pediatricians request specialist consultations according to clinical indications.
6. Nursing observation: The nursing staff monitor interactions between the caregiver and the child during hospitalization, document the observed symptoms and their timing, assess parental behavior, and identify potential patterns (e.g., symptoms manifesting only in the presence of the caregiver). They also ensure the implementation of safety protocols, such as intravenous line protection, and, where legally authorized, video surveillance.
7. Social work assessment: The hospital social workers interview family members, assess psychosocial risk factors (e.g., mental health issues, prior child protection involvement), offer emotional support to the family in a non-accusatory manner, liaise with child protective services, and propose supportive interventions or supervision plans when it is necessary.
8. Blind review: A child neuropsychiatrist independently reviews the patient’s history without knowledge of the initial assessment findings.
9. Forensic evaluation: A forensic pathologist, upon request, contributes by conducting a comprehensive assessment, including a review of medical records and the circumstances surrounding the child’s presentation.

### 2.5. Data Collection

All cases were anonymized and entered into a dedicated spreadsheet-based database. The following variables were recorded:

- Unique identifier for each case;
- Patient age and sex;
- Nationality (place of birth);
- Date of diagnosis;
- Number of emergency department (ED) visits and hospital admissions;
- Presence of chronic or acute medical conditions;
- Clinical specialty responsible for the admission;
- Type and characteristics of perpetrator (social context, substance use, psychiatric disorders);
- Clinical presentations and symptoms;
- Diagnostic classification (Rosenberg criteria);
- Number of red flags (AAP/RCPCH).

Due to the absence of a national electronic medical record system in Italy, complete paper-based hospital chart documentation from prior admissions were examined to verify the diagnosis of MCA. Additional information was obtained by contacting other hospitals when necessary.

### 2.6. Data Analysis

Given the small sample size (n = 8 eight) and the descriptive nature of this case series, statistical analysis was limited to descriptive statistics. Continuous variables are reported as median and range. Categorical variables are reported as frequencies and percentages. Prevalence is reported with 95% confidence intervals calculated using the Wilson score method.

### 2.7. Ethical Considerations

Informed consent was obtained from all subjects involved in the study or their legal guardians. The original and complete datasets are not publicly available because they include personal data of patients protected by the General Data Protection Regulation (GDPR). Requests to access the datasets should be directed to Meyer Children’s Hospital, Florence, Italy.

## 3. Results

### 3.1. Case Identification and Prevalence

During the 12-year study period (2010–2022), the GAIA service evaluated 816 cases of suspected child maltreatment. Of these, 8 cases (0.98%; 95% CI: 0.43–1.94%) were identified as suspected or confirmed MCA.

### 3.2. Demographic Characteristics

The demographic characteristics of the 8 MCA cases are summarized in Table 4.

### 3.3. Perpetrator Characteristics

All perpetrators were biological mothers (8/8, 100%). Perpetrator characteristics are summarized in Table 5.

### 3.4. Healthcare Utilization

Healthcare utilization patterns are presented in Table 6

### 3.5. Clinical Presentations

The frequency of clinical presentations is shown in Table 7.

### 3.6. Diagnostic Classification

Using Rosenberg criteria, cases were classified as follows (Table 8):

All cases met a median of 4 red flags from AAP and RCPCH guidelines (range: 2–5).

### 3.7. Cases Description and Summary

**Case 1.** Female, 4 years at diagnosis, foreign origin. Twenty-three ED (emergency department) attendances from 2016 to mid-2020 for dyspnea, febrile upper-respiratory/otitic illnesses, minor head injuries, reported hematuria, cough, strangury, and wound follow-up; investigations were consistently unremarkable. Concern for medical child abuse (MCA) arose because the older sister (case number 4) was being subjected to MCA by the mother. Rosenberg criteria: possible diagnosis. Four red flags from AAP and RCPCH.

**Case 2.** Male, 4 years at diagnosis, Italian, prior Kawasaki disease at age 2. Five ED attendances in 2020–2021 for fever with rash and cervical swelling, abdominal cramps with suspected parasitosis, pruritus with suspected parasitic infestation, and two minor traumas; investigations were negative. Alleged perpetrator: mother. Rosenberg criteria: possible diagnosis. Three red flags from AAP and RCPCH.

**Case 3.** Female, 4 years at diagnosis, Italian; context of an over-controlling mother and domestic violence against the mother. Four ED attendances in 2021–2022 for rash, fever with vomiting/diarrhea, non-concussive head injury, and otalgia; investigations were negative. Alleged perpetrator: mother. Rosenberg criteria: inconclusive determination. Two red flags from AAP and RCPCH.

**Case 4.** Female, 5 years at diagnosis, foreign origin, sister of Case 1; congenital bilateral thalamo-striatal vasculitis. Thirty-eight ED attendances in 2015–mid-2020 for diverse, recurrent complaints including dyspnea, tremors, inconsolable crying, multiple minor traumas, respiratory and gastrointestinal symptoms, alleged urinary calculi passage, reported gingival “parasite,” palpebral edema, foot laceration, clavicular swelling post-fracture abroad, spontaneous bruising, and torticollis; investigations repeatedly negative. First MCA concern recorded 30 May 2020; taken over by the GAIA Service on 9 July 2020. Alleged perpetrator: mother. Rosenberg criteria: possible diagnosis. Four red flags from AAP and RCPCH.

**Case 5.** Female, 5 years at diagnosis, foreign origin; early-life gliotic parietal lesion, renal asymmetry with reduced left function, neurogenic bladder with recurrent UTIs, renovascular hypertension; socioeconomic adversity and maternal report against nursery. Twenty-one ED attendances between 2005 and 2022 for inconsolable crying, febrile illnesses with respiratory or gastrointestinal symptoms, minor craniofacial trauma, recurrent headaches, suspected maltreatment at nursery, two syncopal episodes, abdominal pain with headache, lower urinary tract symptoms with flank pain, and a classroom panic attack; investigations were negative. Alleged perpetrator: mother. Rosenberg criteria: possible diagnosis. Four red flags from AAP and RCPCH.

**Case 6.** Female, 11 years at diagnosis, Italian; mother of foreign origin. Multiple psychiatric consultations and antipsychotic prescriptions abroad for an ill-defined syndrome led to psychomotor slowing and apathy; after the father discontinued medications in Italy, the child improved and showed no psychosis. MCA by the mother was confirmed. Rosenberg criteria: diagnosis by exclusion. Five red flags from AAP and RCPCH.

**Case 7.** Female, 11 years at diagnosis, Italian; neonatal white-matter micro-infarcts, migraine with tension-type headache and trigeminal neuralgia; multiple allergies and asthma; mother is a nurse. Forty-four ED attendances in 2006–2019 for non-responsive dyspnea without objective exacerbation, multiple minor traumas, febrile illnesses with varied symptoms, headaches resolving with simple analgesia, mucus with blood, low-back pain, transient visual loss with normal ophthalmology, and gingival pain/headache after MRI-related dental appliance heating; investigations were negative. Alleged perpetrator: mother. Rosenberg criteria: possible diagnosis. Four red flags from AAP and RCPCH.

**Case 8.** Female, 12 years at diagnosis, Italian; hypochondriacal syndrome at age 7 following paternal abandonment. Twenty-three ED attendances from 2004 to 2015 for febrile respiratory illnesses, reported blood in stool, multiple soft-tissue swellings, post-mononucleosis abdominal ultrasound requests, contact with infectious diseases, and reported parasite passage; investigations were negative. Alleged perpetrator: mother. Rosenberg criteria: possible diagnosis. Four red flags from AAP and RCPCH.

## 4. Discussion

Medical child abuse (MCA) clearly labels the behavior as abuse and explicitly states the medical connection. It makes no grater claim to a medical diagnosis than do other forms of abuse. Physical or sexual abuse are not medical diagnoses of a specific illness, but rather events in a child’s life that can have medical consequences. The same is true for medical abuse. As an event or series of events, it can be described along a continuum of severity, from mild to moderate to severe. At a certain point along that continuum, as with other forms of child maltreatment, representatives of the community at large determine the need to intervene to protect the child from further harm. All forms of child maltreatment share this property [12,24,25]. Both the American Academy of Pediatrics (AAP) and the Royal College of Paediatrics and Child Health (RCPCH) emphasize that MCA should be framed as child maltreatment, regardless of the caregiver’s intent, focusing primarily on the impact and risk to the child [15,21,26].

Our study identified a prevalence of 0.98% (95% CI: 0.43–1.94%) of MCA among child maltreatment cases at a tertiary pediatric hospital over a 12-year period. This finding is consistent with the literature reporting MCA as a rare but serious form of child abuse, with estimated incidence rates of 0.5 to 2 per 100,000 children annually [15,16,27,28]. This figure is also consistent with a previous Italian study from Rome (0.53%) based on a similarly sized sample (n = 751) [27].

Our findings echo trends in four major case series: Schreier and Libow (1993) [24], Rosenberg (1987) [5], Sheridan (2003) [17], and Meadow (1995) [15], which report mean victim ages between 2.8 and 4.0 years, with female victims comprising 54–60%, and maternal perpetrators in 96–100% of cases. The median age at diagnosis in our cohort was 5 years (range: 4–12 years), which aligns with published data indicating that most victims are under 6 years old. The female predominance (75%) in our sample is notable, though the small sample size limits definitive conclusions. The literature reports variable sex distributions, with some studies showing no clear predominance [12,13].

Consistent with the literature, all perpetrators in our series were biological mothers (100%), matching the reported 85–95% maternal perpetration rate in confirmed MCA cases [11,12,13,14]. One perpetrator (12.5%) had medical training as a nurse, consistent with reports that perpetrators often have some familiarity with medical systems [15]. The presence of psychosocial stressors, including domestic violence, social isolation, and psychiatric history, aligns with known risk factors for perpetrators [16,17].

The 2022 systematic review by Abdurrachid and Gama Marques [29], which synthesized 108 articles and 81 case reports over 15 years, further supports these trends—reporting 91% female perpetrators, 17% healthcare workers, and most victims under 6 years old. Three out of our eight cases (37.5%) involved single mothers, while several others revealed psychosocial stressors such as domestic violence, precarious finances, or previous complaints filed with child protective services. Compared to Abdurrachid and Gama Marques’s findings, our data showed a slightly lower proportion of healthcare worker perpetrators (12.5%) and an older average victim age. These differences could reflect geographic, cultural, or systemic variations in case identification.

International data indicate that neurological (40–60%), gastrointestinal (30–45%), and respiratory (20–35%) symptoms are the most commonly falsified or induced. In our series, the most frequent presentations included the following:

- Infectious diseases (21.6%);
- Exaggeration of trauma (18.9%);
- Neurological symptoms, primarily headaches (10.8%);
- Skin and abdominal complaints;
- Respiratory issues, notably dyspnea.

These categories align closely with international data, where symptoms are typically subjective, non-specific, and difficult to confirm clinically. Fabricated symptoms such as vomiting, dyspnea, or seizures—often reported only by the caregiver—were widely observed in our cohort, consistent with literature noting the frequent tampering with samples, symptom induction, or misleading history [18,30,31,32].

The median of 23 ED visits (range: 4–44) before diagnosis underscores the prolonged nature of MCA and the challenge of early detection, matching data from Sheridan et al. [17] who report 15–25 clinical encounters before MCA is suspected. Abdurrachid and Gama in a 2022 review [29] confirm that repeated and unnecessary interventions, including hospital admissions and surgeries, are common before the pattern of abuse becomes evident.

We found that 62.5% of children had pre-existing or chronic medical conditions (e.g., neurological deficits, renal disease, psychiatric disorders), mirroring global findings that complex medical histories often provide a convenient setting for symptom manipulation [33,34,35].

Using Rosenberg criteria, only 1 case (12.5%) met criteria for “definitive diagnosis,” while the majority (62.5%) were classified as “possible diagnosis.” This distribution highlights the inherent diagnostic challenges in MCA, where definitive proof of fabrication or induction is often difficult to obtain. The reliance on circumstantial evidence, pattern recognition, and exclusion of alternative explanations necessitates a cautious, multidisciplinary approach [19].

The detection of MCA remains complex and delayed, often due to fragmented care, lack of shared records, and reliance on the caregiver’s narrative. All cases met a median of 4 AAP/RCPCH red flags (range: 2–5), demonstrating the utility of these alerting signs in raising clinical suspicion. However, the presence of red flags alone is insufficient for diagnosis and must be interpreted within the broader clinical context [7,8].

These patterns were strongly represented in the international case series and in the 2022 review [29], which also identified caregiver eagerness for medical interventions and manipulation of medical data as frequent traits [36,37]. In Case 6, we observed a symptom resolution during periods of custody with the father—an essential diagnostic clue also highlighted in Meadow’s observations [15].

The GAIA service’s multidisciplinary approach, involving pediatricians, forensic pathologists, psychiatrists, psychologists, nurses, and social workers, reflects best practices outlined in international guidelines [7,8,25]. The standardized nine-step protocol ensures systematic evaluation, reduces diagnostic bias, and facilitates comprehensive documentation, all critical elements in MCA management.

The inclusion of forensic professionals is particularly valuable in documenting inconsistencies, guiding legal pathways, and differentiating genuine pathology from fabricated symptoms [30,36]. The blind review by an independent child neuropsychiatrist adds an additional layer of diagnostic rigor, helping to minimize confirmation bias

Italy’s regulatory framework prohibits covert video surveillance (CVS) in hospital settings without judicial authorization [38], in contrast to UK and US protocols where CVS is ethically approved under strict conditions for severe cases [39]. This limitation underscores the critical role of medical documentation in Italy. Meticulous and longitudinal records could allow clinicians to identify patterns across multiple encounters and build a timeline of symptom discrepancies, often the most reliable diagnostic tool in our context.

A recent national survey found that only 2% of Italian pediatricians contacted judicial authorities when suspecting MCA, often preferring to speak directly with the caregiver [40]. This reluctance mirrors concerns raised in a 2022 case series with review [41] about underreporting, inadequate follow-up, and lack of legal action in many confirmed cases. The same article notes that abuse resulted in death in 6–10% of cases, with long-term psychological harm common among survivors [41].

Fortunately, no fatalities occurred in our sample. However, long-term outcomes could not be assessed due to fragmented follow-up data, a significant limitation of this study. Literature suggests that up to 30–50% of affected children develop lasting psychological or developmental issues, and some may later reenact FDIA behaviors themselves [42,43,44]. Systematic long-term follow-up of MCA victims is essential to understanding the full impact of this form of abuse and to guide intervention strategies.

Our study compared with literature review highlight that early detection of MCA requires the following:

1. High index of suspicion: Clinicians should be alert to red flags, particularly unexplained or inconsistent symptoms, excessive healthcare utilization, and symptom resolution when the child is separated from the caregiver.
2. Thorough documentation: Meticulous record-keeping is essential, including detailed descriptions of reported symptoms, objective findings, caregiver behavior, and response to interventions.
3. Multidisciplinary collaboration: No single clinician can diagnose MCA alone. A team-based approach ensures comprehensive evaluation and reduces diagnostic error.
4. Access to shared medical records: The absence of a national electronic medical record system in Italy significantly hampers MCA detection. Expanding access to unified electronic medical records would facilitate pattern recognition across multiple healthcare encounters and institutions.
5. Training and education: Healthcare professionals require training in recognizing and managing MCA, including understanding legal and ethical obligations for reporting suspected abuse.

The study has some limitations. The small sample size (n = 8), single-center design, and retrospective nature limit generalizability and statistical power. Reliance on existing medical records may result in incomplete or inconsistent data, introducing documentation bias and potentially missing cases with inadequate records. Cases were identified through referral to the GAIA service, potentially representing only the most severe or obvious cases (selection bias). The true prevalence of MCA is unknown, and lack of long-term follow-up precludes assessment of lasting outcomes. Inter-rater reliability data were not formally assessed.

Further research should focus on multicenter studies to better characterize MCA epidemiology and clinical features, both in Italy and internationally. Prospective studies with standardized data collection would improve data quality and enable more robust analysis. Long-term follow-up may help in understanding long-term outcomes and guiding intervention strategies. The development of validated screening tools could help clinicians in identifying high-risk cases earlier. Intervention studies on the effectiveness of different intervention strategies for both victims and perpetrators and the implementation of electronic health records, at least on a national level, would significantly enhance MCA detection and management.

## 5. Conclusions

Medical child abuse, although rare (0.98% of child maltreatment cases in our series), represents a potentially severe and underdiagnosed form of child maltreatment. Its detection is hindered by fragmented care, lack of shared records, and caregiver deception. Multidisciplinary collaboration—particularly involving forensic pathologists—plays a pivotal role in diagnosis and legal management. Our findings highlight the importance of structured documentation and inter-institutional data sharing. The GAIA multidisciplinary model offers a replicable framework for the effective management of suspected MCA cases. Expanding access to unified electronic medical records, as advocated by national guidelines, and implementing multicentric research initiatives are essential steps toward improving recognition, reporting, and outcomes for affected children.

Healthcare professionals must maintain a high index of suspicion for MCA in cases of unexplained or inconsistent symptoms, excessive healthcare utilization, and symptom patterns which change with caregiver presence. Early detection and intervention are critical to preventing ongoing harm and improving outcomes for vulnerable children.

## Figures and Tables

**Table 1:** Perpetrator characteristics according to literature concerning MCA.

Category	Common Risk Features
Psychiatric disorders	Personality disorders (especially borderline or histrionic), depression, and somatoform disorders [14]
History of abuse	Personal history of childhood abuse or neglect, especially unresolved trauma
Need for attention	Desire for sympathy, praise, or validation from healthcare professionals
Social isolation	Lack of social support or limited external family contact
Medical knowledge	Often has some training or familiarity with medical systems (e.g., nurse, caregiver, or frequent hospital visitor) [15]

**Table 2:** Victim characteristics according to literature concerning MCA.

Factor	Details
**Age**	Most victims are under 6 years old; infants and toddlers are most at risk [18]
Medical complexity	Children with genuine chronic illnesses may become targets, as their condition provides cover for abuse
Communication barriers	Non-verbal, developmentally delayed, or pre-verbal children are more vulnerable
Passive temperament	Submissive or easily controlled children are often selected

**Table 3:** Summary of the key diagnostic red flags reported by AAP and RCPCH.

Clinical Indicators	Examples
Symptoms only witnessed by caregiver	Seizures, apnea, bleeding
Symptoms disappear in hospital	Child improves during admission
Inconsistent medical history	Contradictory accounts from caregiver
Multiple medical providers	Frequent hospital visits or “doctor shopping”
Excessive medical interventions	Unnecessary surgeries, diagnostic tests
Unusual laboratory findings	Tampering with samples (e.g., blood in urine)

**Table 4:** Demographic characteristics of MCA victims (n = 8).

Characteristic	Value n (%)
Age at diagnosis	
Median (range) in years	5 (4–12)
[Mean ± SD]	7.0 ± 3.2
Sex	
Female	6 (75.0)
Male	2 (25.0)
Nationality	
Italian	4 (50.0)
Foreign origin	4 (50.0)
Pre-existing medical conditions,	
Yes	4 (50.0)
No	4 (50.0)

**Table 5:** Perpetrator characteristics (n = 8).

Characteristic	n (%)
Relationship to victim	
Biological mother	8 (100.0)
Medical background	
Healthcare worker (nurse)	1 (12.5)
No medical training	7 (87.5)
Psychosocial factors	
Domestic violence context	1 (12.5)
Social isolation	3 (37.5)
Documented psychiatric history	2 (25.0)

**Table 6:** Healthcare utilization patterns (n = 8).

Variable	Value
Emergency department visits	
Median (range)	23 (4–44)
Mean ± SD	22.6 ± 13.8
Time to diagnosis	
Median duration of abuse before detection (years)	>1 year (all cases)

**Table 7:** Frequency of clinical presentations (n = 8).

Presentation	n (%)
Minor trauma	7 (87.5)
Fever	6 (75.0)
Respiratory complaints (dyspnea, cough)	5 (62.5)
Gastrointestinal symptoms (abdominal pain, vomiting, diarrhea)	4 (50.0)
Headaches	3 (37.5)
Dermatological complaints	3 (37.5)
Urinary symptoms	2 (25.0)
Neurological symptoms (seizures, syncope)	2 (25.0)
Reported bleeding (hematuria, blood in stool)	2 (25.0)

**Table 8:** Diagnostic classification using Rosenberg criteria (n = 8).

Classification	n (%)
Possible diagnosis	5 (62.5)
Definitive diagnosis	1 (12.5)
Diagnosis by exclusion	1 (12.5)
Inconclusive determination	1 (12.5)

## Data Availability

The original and complete datasets presented in this article are not readily available because they include personal data of patients protected by GDPR. Requests to access the datasets should be directed to Meyer Children Hospital, Florence.
